# Next generation sequencing for neurological diseases: New hope or new hype?

**DOI:** 10.1016/j.clineuro.2012.09.030

**Published:** 2013-07

**Authors:** M.J. Keogh, P.F. Chinnery

**Affiliations:** Mitochondrial Research Group, Newcastle University, UK

**Keywords:** Exome, Next-generation, Sequencing, Mendelian, Mutation

## Abstract

Over the past year huge advances have been made in our ability to determine the genetic aetiology of many neurological diseases through the utilisation of next generation sequencing platforms. This technology is, on a daily basis, providing new breakthroughs in neurological disease. The aim of this article is to clearly describe the technological platforms, methods of data analysis, established breakthroughs, and potential future clinical and research applications of this innovative and exciting technique which has relevance to all those working within clinical neuroscience.

## 1 Introduction

Progress in our understanding of the genetic basis of neurological disease has expanded rapidly over the past 20 years. Whilst it is now clear that there is an extensive spectrum of genetic involvement in the aetiology of neurological disease, the common paradigm employed in most studies has been to essentially divide the disorders into two dichotomous groups. Firstly, ‘common’ neurological diseases with complex phenotypes and a probable multigenic component to their aetiology (such as Parkinson's disease, multiple sclerosis and epilepsy), and secondly ‘rare’ neurological diseases which are perceived to have a more narrow phenotype and obey strict Mendelian laws of inheritance.

For the investigation of many common, complex diseases, candidate gene and subsequent genome-wide association studies (GWAS) have been widely adopted identifying an extensive list of loci associated with neurological disease [1–6]. Such studies have assisted in determining mechanisms of disease, however, most identified alleles are predicted to have a very small effect size [7], and infrequently lead to the discovery of causal polymorphisms [8].

Approaches to identify the genetic basis of rare Mendelian disorders have advanced slowly but surely over the past decade, being largely based on well-established techniques such as positional cloning and linkage analysis followed by targeted candidate gene screening. Cloning genes were critically dependent on identifying large well-characterised pedigrees, groups of pedigrees with a presumed identical genetic aetiology, or consanguineous families with a disease likely due to homozygous mutations. To some extent, finding the gene was a matter of “luck”, based on the pedigree structure, compounded by the cost and physical time taken to sequence a large number of genes, which severely limited progress. As a result the genetic basis of less than 50% of all Mendelian disorders has been determined [9], and this has particular relevance in neurology. For example, in the USA over half of patients recently recruited to a national clinical programme tasked with determining the genetic basis of rare diseases have neurological disorders; by far and away the more prevalent specialty [10]. Our own observations echo this estimation, with approximately half of the patients in our neurogenetic clinic having no confirmed molecular diagnosis (*unpublished observations*).

However, this last year has seen a paradigm shift in the investigation of these rare Mendelian disorders, largely based on the technical advance of a new DNA sequencing technology termed ‘next generation sequencing’ (NGS), also known as deep resequencing, or massively parallel sequencing, which is revolutionising the investigation of rare disorders [11]. NGS may also enable the elucidation of the contribution of rare alleles in common disorders, potentially offering significant breakthroughs in our understanding.

The aim of this article is to review the most widely used approach, its role for rare Mendelian neurological disorders, and its potential for wider use across the continuum of neurological disease.

## 2 A new technique

Next generation sequencing has been evolving since 2005 [12] and provides the ability to dramatically increase the speed at which DNA can be sequenced at a fraction of the cost of older sequencing technology. To illustrate this, in 2001 the Human Genome Project used first generation Sanger sequencing technology to sequence the human genome, taking 13 years and $2.7 billion to achieve its goal [13–15]. Next generation sequencing can now sequence an individual genome in under 2 weeks for approximately US$ 4000, representing remarkable progress.

However, based on current thinking, the whole genome does not need to be sequenced to identify most human disease genes. Eighty-five percent of pathogenic mutations causing Mendelian disorders are found within the segments coding for proteins (exons) [16], which collectively are referred to as the “human exome” [17]. This dramatically cuts down the region that needs to be sequenced in patients and families with undiagnosed neurogenetic disorders, reducing the cost and time to approximately $1500 and 48 h respectively. However, as discussed later, it is the bioinformatic processes and data analysis of the DNA sequences which now predominantly influence the time taken to achieve a definitive result.

## 3 So how does next generation sequencing work?

**Fig. 1:**
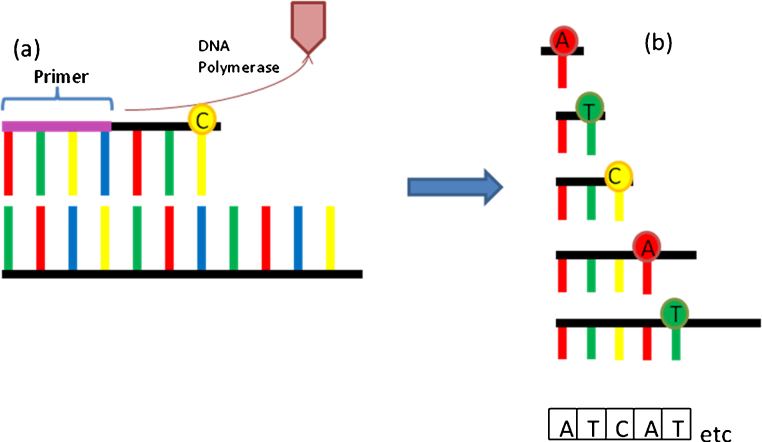
(a) A DNA primer anneals to the template sequence of DNA. DNA polymerase binds incorporating free nucleotides into the DNA strand. The strand elongates until randomly a fluorescent labelled nucleotide in incorporated, and its chemical alteration results in termination of the DNA strand. (b) DNA strands are read in order of chain length (top to bottom in diagram) and the sequence is therefore determined (ATCAT).

DNA sequencing was first developed in 1975 by Sanger and Coulson [18] and his techniques are still used widely today. This ‘Sanger sequencing’ or first generation sequencing, is based on the application of oligonucleotide primers on either side of the desired DNA sequence followed by the addition of DNA polymerase and a mixture of nucleotide “building blocks” enabling multiple copies of the original DNA sample to be generated. The introduction of chain terminating nucleotides in 1977 [19] enabled the generation of a whole array of different copies of the original DNA sequence “chain terminated” at all possible lengths, which are then separated out on gel or capillary system by electrophoresis. Using known specific labelled nucleotides (A, C, T or G) it is possible to assemble the original DNA sequence (Fig. 1).

Next generation sequencing is based on the principle of massively parallel sequencing. This essentially means that thousands or millions of pieces of DNA can be sequenced at the same time.

**Fig. 2:**
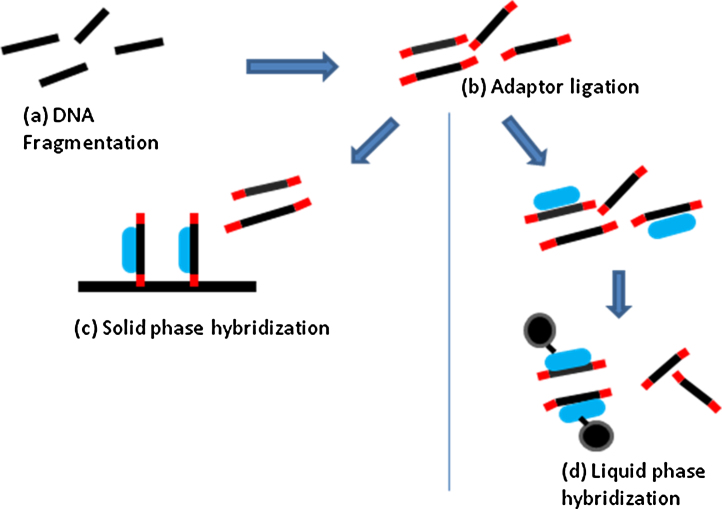
An example of two different DNA enrichment approaches though numerous methods are available (a) DNA is randomly fragmented (black lines). (b) Adaptors are ligated to each end of the DNA (red lines). (c) Solid phase hybridisation occurs on a DNA microarray. A collection of DNA spots/bait probes (blue line) bind to DNA regions from the exome but not intronic fragments of DNA which are washed away. Thereafter the exomic DNA is eluted. (d) Liquid phase hybridisation. DNA probes (blue lines) attach to DNA fragments from the exome. Thereafter, streptavidin beads (black and grey circles) are added to allow physical separation. (For interpretation of the references to color in this figure legend, the reader is referred to the web version of the article.)

Initially DNA is fragmented into multiple short segments called a shotgun library. Adaptors are ligated to the ends of each fragment. These adaptors are short sequences of DNA which have priming sites within them for the subsequent amplification steps. The segments of DNA (complete with adaptors) are then mixed with probes which correspond to regions within the exome. The shotgun library is then ‘enriched’ for the sequences of interest, using beads or a solid plate to allow physical separation of the exome from remaining DNA, and this is washed away. Custom arrays can be designed to enrich for specific groups of genes of interest, the whole exome, and exon-flanking regions (Fig. 2).

**Fig. 3:**
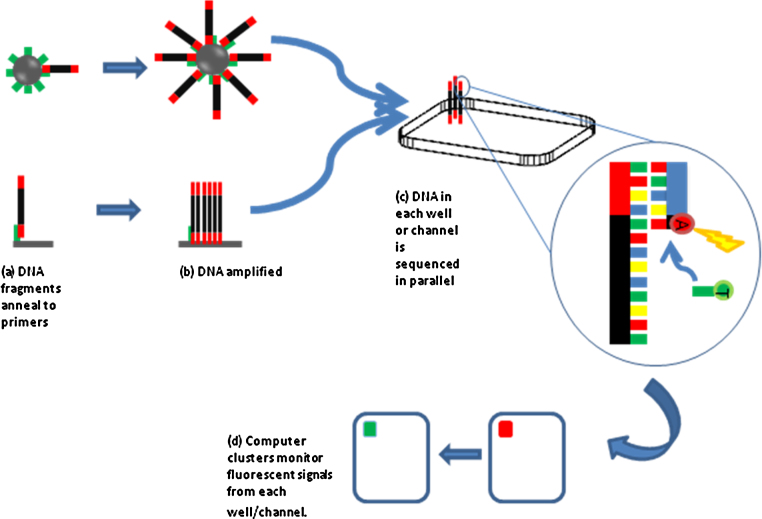
Two common forms of DNA sequencing are shown. The process for one DNA fragment only is shown for clarity. (a) Top panel – DNA adaptors on a DNA fragment bind to a complementary primer on a bead (bead – grey circle, primer – green line) which represents the 454 technique. (a) Bottom panel – DNA fragments are passed over a lawn of primers where they attach which represents the illumina (Solexa) technique. (b) In top and bottom panel, DNA is amplified many times so that multiple copies of the fragment are on the bead (top panel) or in a cluster (bottom panel). The fragments are copied numerous times on each bead before being filtered and the DNA denatured to a single strand and placed into a specific well (for the 454 technique), or remain in a specific channel on a slide for the illumina method. (c) Sequencing primers, polymerase and nucleotides are added to the mix. As each nucleotide is added a laser activates a fluorescence, which is represented by incorporation of an adenine nucleotide and soon to be followed by a thymidine nucleotide. (d) Sensors detect the colour change from each well or cluster with each nucleotide addition and the sequence read. (For interpretation of the references to color in this figure legend, the reader is referred to the web version of the article.)

Following DNA enrichment, several manufacturers, each with differing techniques provide next generation sequencing platforms, the individual merits of which have been extensively reviewed elsewhere [12]. Demonstrated in Fig. 3, however are two of the most common forms of DNA sequencing technique, the illumina (previously known as Solexa) and 454 method (also known as Roche FLX) though several more exist. Briefly, the 454 method anchors DNA fragments to resin beads for amplification before transferring them into wells on a plate together with enzyme beads for sequencing. The illumina method binds fragments to a slide where DNA is amplified in clusters before sequencing takes place. Several other methods also exist, and the techniques vary in DNA fragment read length, time, cost and potential applications.

Once each segment of DNA is amplified and in situ on the slide or plate, florescent nucleotides are added, together with DNA polymerase and sequencing primers. As fluorescent tagged bases are incorporated to each strand on each bead or channel, in real time, laser activation of the fluorescence can be read. Computers monitor each cluster, and can determine the sequence of many clusters at the same time (Fig. 3), and hence how sequencing data can be generated at such a phenomenal rate.

## 4 Data processing

Whilst huge quantities of sequencing data can be produced relatively quickly, the data analysis can be lengthy and difficult. Taking an individual case, whole exome sequencing will yield around 20,000–25,000 single nucleotide polymorphisms (SNPs) [20], and determining which, if any represent a pathogenic mutation is difficult. The approach to date has been for the sequences to first undergo a process called ‘discrete filtering’. This entails cross-referencing the 20,000–25,000 or so polymorphisms with a set of ‘controls’, i.e. exome data from unaffected individuals. Such control data is publically available on databases such as dbSNP [21] and the 1000 genomes project [22], together with growing individual ‘in house’ exome control data sets generated within centres using exome sequencing. This step helps to filter out polymorphisms present within the local population, many of which are not recorded on public database. Cross referencing the patients SNPs with those in publically available databases assumes that mutations seen in the control data are not pathogenic (see later section for how this may not be true), removing approximately 97–98% of the polymorphisms, and leaving roughly 500–700 remaining [20,23].

The next step is to further filter these remaining mutations. Firstly, those that do not fit with the mode of inheritance (e.g. heterozygous mutations in a presumed homozygous recessive disease) are removed, and then mutations are examined which occur in genes known or predicted to have involvement in the biological process or diseases with a similar phenotype. Candidate mutations which occur in a patient can also be checked to see if they segregate with disease phenotype in other family members, assuming appropriate samples are available. An additional stage often involves the utilisation of new software tools which are now widely available and help to determine which mutations may have functional impact on the transcribed gene, are likely to be pathogenic mutations, or are polymorphisms with no effect [24–26]. These packages assist with the filtering process, however their results still need to be interpreted in conjunction with the pedigree information and biological significance, and are not infallible.

The above description is an outline of the process for each individual. Usually, several members of a family (affected and unaffected) will undergo exome sequencing when searching for a novel pathogenic mutation. For example sequencing two distantly related relatives (e.g. cousins) within a phenotype, and cross-referencing their data can significantly limit the potential candidate mutations to an even smaller number.

## 5 Problems

There are of course limitations with exome sequencing, and they can best be divided into ‘hardware’ and ‘software’ problems. The main ‘hardware’ problems arise through the incomplete enrichment of the sequence of interest. For “whole exome” sequencing, coverage rates have improved from the 92% seen in initial studies [27], but achieving 100% coverage of the entire exome is still not possible. Secondly, it must be appreciated that coverage is not a dichotomous concept. Coverage can be quantified in terms of depth, which is the average number of times a given DNA nucleotide is represented in sequence reads. If coverage is low, i.e. a particular base is only covered 1 or 2 times/in 1 or 2 reads, then any sequencing error at that point could be interpreted as a mutation, or alternatively a genuine mutation could be missed [28]. Thus, as part of the sequencing analysis it is important to be able to assess the exome for regions of poor coverage which may confound results.

Thirdly, to run the assay, several micrograms of DNA are needed. Whilst this is easily obtainable from a few millilitres of blood, extracting enough DNA from limited and finite volumes of stored tissue such as brain can prove difficult. Finally, and perhaps obviously, whole exome sequencing only sequences the exome, hence pathogenic mutations in non coding regions which may include vital regulatory regions and splice sites can exist and go undetected with exome sequencing. Such mutations are estimated to cause disease in around 15% of all Mendelian disorders [16], and at present whole genome sequencing would be needed to assist in detecting these mutations.

Software, or ‘analytical’ challenges include difficulty in detecting mutations such as repeat expansions. Despite recent studies in amyotophic lateral sclerosis-frontotemporal dementia (ALS-FTD) in which novel repeat expansions were detected with NGS [29], exome sequencing is generally not useful for identifying repeat expansions [23]. This limitation is particularly important in neurology with many conditions such as Huntington's disease, myotonic dystrophy, Friedreich's ataxia and forms of spinocerebellar ataxia occurring due to trinucleotide repeat expansions [30,31] and hence disorders with a similar genotypic abnormality may not be detected with exome sequencing.

Secondly, although millions of sequences of DNA are produced, they are relatively short, generally in the order of 25–350 bases in length [12]. This creates problems when processing short reads as many short sequences of DNA repeat themselves throughout the genome, and hence they may be able to align at several positions. In fact, about 15–20% of the short DNA reads cannot be unambiguously mapped to a single location in the genome [32].

Thirdly, small base substitutions or insertions/deletions may be missed if the DNA in the sequence is slightly misaligned to the reference genome. Alignment difficulties can also create the impression of new mutations that are not actually present in the original DNA sequence [20].

Finally, several pathogenic mutations are also present on publically available “control” databases, and may be filtered out of the analysis at an early stage, “throwing out the baby with the bathwater”. These variants are present in the database for several reasons. For example, many neurogenetic disorders present late in life, or the diseases may not be fully penetrant and therefore younger patients or those who have not yet manifested the phenotype may be included in reference data.

## 6 How may exome sequencing help with neurological disease?

In a research setting, exome sequencing is a potentially revolutionary tool for the investigation of rare Mendelian disorders. Putting the technical advantages already discussed aside, a key strength of this technique is that it is able to detect new mutations in an unknown gene in very small family pedigrees, and sometimes only on one affected individual with a recessive disease. This simply would not have been possible in the relatively recent past because several affected individuals were needed to define a disease locus through genetic linkage or homozygosity mapping. Finally, exome sequencing can detect mutations in unexpected genes, broadening the phenotype of a particular genetic disorder. This is not possible with the candidate-gene based approach to diagnostics.

Data highlighting such advantages for neurological disease are rapidly accumulating. For example, Wang et al. discovered a novel mutation in the *TGM6* gene as a cause of spinocerebellar ataxia through exome sequencing. Linkage analysis using over 20 members of the family had identified a relatively large region, consisting of 91 genes in which the mutation was likely to be present. Whole exome sequencing of just 4 patients in the family was able to identify the mis-sense mutation in the *TGM6* gene [33]. For amyotrophic lateral sclerosis (ALS), exome sequencing with small familial cohorts has also lead to revolutionary developments in our understanding of the disease. In a study of 2 affected members of an Italian family with an autosomal dominant pedigree of ALS, a mutation of the VCP gene was identified and speculated to account for 1–2% of all familial ALS [34]. More recently, the use of next generation sequencing lead to the finding of a hexanucleotide repeat expansion in the *C90RF72* gene, which the authors estimated to be responsible for over 80% of familial ALS in Finland, and potentially 21% of sporadic ALS [29]. This highlights how using next generation sequencing in small familial cohorts can lead to dramatic advances for more prevalent diseases.

Exome sequencing for extremely rare neurological conditions (for example in which patients are isolated cases in their families) has also been successful. Mutations in *POLR3A* and *B* encoding RNA polymerase subunits for example were shown to be the cause of autosomal-recessive hypomyelinating leukoencphalopathy in a study of 3 unrelated affected individuals [35]. Additionally, while not being able to find a causative mutation, exome sequencing of 4 unrelated individuals with monomelic amyotrophy showed that variants of 2 genes (KIAA1377 and *C5ORF42*) increased the risk of the disease significantly (OR = 61.69) [36]. In non-neurological conditions, 4 unrelated patients suffering from Schinzel–Giedion syndrome, and 10 unrelated patients with Kabuki syndrome enabled the identification of novel mutations causative of these conditions [37,38]. The difficulty however in using non-related individuals is that there is significant genetic heterogeneity between patients making it more difficult to narrow the filter to a causative mutation as discussed earlier. Additionally, whilst patients with neurological conditions from different families may be phenotypically similar, there is no guarantee they will harbour the same mutation, or even that it will lie in the same gene, and therefore looking for concordant mutations may lead to inaccurate results.

The ability of the technique to detect mutations which were incongruous to the clinical phenotype is highlighted in a recent study by Montenegro et al. In this study, exome sequencing was performed on two affected family members with an atypical phenotype of Charcot–Marie–Tooth disease (CMT). There was reported male–male inheritance in the family pedigree, however exome sequencing revealed an X-linked form of CMT (*GJB1* mutation), whilst simultaneously excluding over 30 other known mutations causing CMT [39].

## 7 Research applications for non Mendelian disorders

Whilst exome sequencing is now at the fore of research into rare Mendelian disorders, it is also likely to find a key role in the investigation of common, sporadic neurological conditions. Genome-wide association studies (GWAs) over the last 5–7 years have been the ‘modus operandi’ of research in this area, and have offered great insight into common variants associated with neurological diseases such as multiple sclerosis, Alzheimer's disease, and epilepsy [1,40–44]. GWA studies involve testing for up to a million or more specific SNPs in each genome. The results are then compared between cohorts of affected individuals and unaffected controls to see if any polymorphisms correlate with the disease and hence determined to be susceptibility loci. More recently, the focus of attention has been sub-phenotypes of disease, providing the studies are adequately powered to address these issues. Using whole exome sequencing it is possible to determine whether rare alleles with a larger effect size contribute to the genetic landscape of common diseases [45,46]. Truly large scale exome studies have not yet been published, but the application in sporadic disease has already been shown, where 20 unrelated patients with autism and their parents were exome sequenced, identifying 21 de novo mutations, 11 of which were protein altering, offering new candidate genes for the condition [47]. The importance of such studies are that they may be able to detect low frequency variants with a high relative risk, and additionally assist in characterising the regions previously suggested to be associated with disease from GWA studies [48]. However, as they are likely to detect de novo mutations, huge numbers of samples will need to be included in such studies to prove causation, and sequencing trios (i.e. an affected individual and their parents) will add to the cost.

## 8 Clinical applications?

There are also several potential clinical applications of exome sequencing which are likely to be seen in neurological practice within the next few years. The first of these is the potential ability to replace certain ‘panels’ of molecular genetic assays. For example, the use of exome sequencing has recently been shown to be suitable to screen for the known mutations associated with hereditary spastic paraplegias (HSP) and a selection of muscle disorders [49]. Similarly, for CMT, the ability with one sequencing run to test for all known mutations (which currently number over 30) may have excellent diagnostic and financial benefits [39].

Whilst exome sequencing at present may only be suitable for screening phenotypes associated with significant genetic heterogeneity (such as CMT or HSP), with the number of genes associated with numerous neurological conditions continuing to grow, testing by way of exome sequencing may become common. However, caveats to this apply. As discussed earlier, exome sequencing at present is not suitable to detect trinucleotide repeats, and hence has limited value in detecting conditions such as myotonic dystrophy, fragile X-syndrome, and some forms of spinocerebellar ataxia for example. It also has variable ability to detect copy number variations, hence having limited function to assess the *PRKN* and *SNCA* genes in Parkinson's disease [48,50].

An additional diagnostic benefit is the ability to store the exome sequencing data, and hence test for new mutations as they are discovered, negating the need to repeatedly obtain and re-process patient samples. However, the storage of highly detailed genetic data also poses legal, logistical and ethical challenges. For example, exome sequencing has already discovered mutations which were not the desired focus of investigation when a mutation causing ciliary dyskinesia was discovered in a family undergoing exome sequencing in investigation of Miller syndrome [51]. How we consent and inform patients of the potential for such incidental findings is a clinical and ethical challenge. Consideration of the consent process to cover such situations in conjunction with the ethical issues regarding the storage and sharing of highly personal information needs careful consideration [52].

## 9 Conclusion

Exome sequencing is an exciting and extremely powerful technique which is currently revolutionising research in rare Mendelian disorders. Its use is likely to eventually encompass the investigation of sporadic and common diseases, leading to the detection of new causative mutations and association alleles which will expand our understanding of neurological disease. Whilst whole genome sequencing is likely to challenge its place at the fore of neurogenetics over coming years, the basic science and clinical information learnt from exome sequencing may rapidly reshape research paradigms and facilitate future developments with whole genome sequencing.
